# Genome Sequence of a Clinical Isolate of the Human Pathogenic Strain “*Candidatus* Borrelia fainii” Qtaro

**DOI:** 10.1128/mra.01318-22

**Published:** 2023-04-19

**Authors:** Kentaro Itokawa, Kozue Sato, Yongjin Qiu, Jyunya Yamagishi, Katendi Changula, Naoko Kawai, Kyoko Hayashida, Joseph Ndebe, Bernard Mudenda Hang’ombe, Hirofumi Sawa, Shinji Kasai, Yukihiro Akeda, Hiroki Kawabata

**Affiliations:** a Department of Medical Entomology, National Institute of Infectious Diseases, Shinjuku, Tokyo, Japan; b Department of Bacteriology I, National Institute of Infectious Diseases, Shinjuku, Tokyo, Japan; c Management Department of Biosafety, Laboratory Animal, and Pathogen Bank, National Institute of Infectious Diseases, Shinjuku, Tokyo, Japan; d Department of Virology I, National Institute of Infectious Diseases, Shinjuku, Tokyo, Japan; e Division of International Research Promotion, International Institute for Zoonosis Control, Hokkaido University, Kita-ku, Sapporo, Japan; f Division of Collaboration and Education, International Institute for Zoonosis Control, Hokkaido University, Kita-ku, Sapporo, Japan; g Department of Paraclinical Studies, School of Veterinary Medicine, University of Zambia, Lusaka, Zambia; h Department of Disease Control, School of Veterinary Medicine, University of Zambia, Lusaka, Zambia; i Africa Centre of Excellence for Infectious Diseases of Humans and Animals, University of Zambia, Lusaka, Zambia; j Division of Molecular Pathobiology, International Institute for Zoonosis Control, Hokkaido University, Kita-ku, Sapporo, Japan; k One Health Research Center, Hokkaido University, Kita-ku, Sapporo, Japan; l Global Virus Network, Baltimore, Maryland, USA; m International Collaboration Unit, International Institute for Zoonosis Control, Hokkaido University, Kita-ku, Sapporo, Japan; n Hokkaido University, Institute for Vaccine Research and Development, Kita-ku, Sapporo, Japan; Indiana University, Bloomington

## Abstract

We report sequences of the complete linear chromosome and five linear plasmids of the relapsing fever spirochete “*Candidatus* Borrelia fainii” Qtaro. The chromosome sequence of 951,861 bp and the 243,291 bp of plasmid sequences were predicted to contain 852 and 239 protein-coding genes, respectively. The predicted total GC content was 28.4%.

## ANNOUNCEMENT

Originally isolated from patient blood in 2017 in Zambia, “*Candidatus* Borrelia fainii” strain Qtaro was identified as relapsing fever *Borrelia* by housekeeping gene sequencing (GenBank accession number LC382043) (1). Here, we report the linear chromosome and five linear plasmid sequences from a culture of “*Ca.* Borrelia fainii” strain Qtaro. This genome sequencing study did not require approval by the institutional ethics committee. A low-passaged culture of “*Ca.* Borrelia fainii” strain Qtaro was grown in modified Barbour-Stoenner-Kelly medium at 34°C (2), and the cell pellet was harvested by centrifugation at 5,000 × *g* and 4°C. Total genomic DNA (gDNA) was extracted using the Monarch high-molecular-weight (HMW) DNA extraction kit (New England Biolabs, Ipswich, MA). To cleave the hairpin structure at the telomeres of genome and linear plasmids (3), 1 μg of gDNA was digested with 90 U of S1 nuclease (TaKaRa Bio, Shiga, Japan) at room temperature (RT) for 15 min and then purified using the AMPure XP system (Beckman Coulter, CA, USA). A next-generation sequencing (NGS) library was constructed for Nanopore from the S1 nuclease-treated (S1 library) and untreated (non-S1 library) gDNA (neither sheared nor size selected) using the LSK-110 kit (Oxford Nanopore Technologies, Oxford, UK), sequenced in a R.9.4.1 flow cell using the MinION instrument, and subsequently base called using Guppy version 6.1.2 (Oxford Nanopore Technologies) with a super accuracy model. In total, 244 Mb (*N*_50_, 27 kb) and 639 Mb (*N*_50_, 24 kb) of sequence reads were obtained from the S1 and non-S1 libraries, respectively. Reads less than 1,500 bp were discarded, and adapters were trimmed using Porechop (https://github.com/rrwick/Porechop).

Reads from the S1 library were used to *de novo* assemble the chromosome (952 kb) using Flye version 3.9.7 (4). All reads from linear plasmids were subjected to an all-versus-all homology comparison using Minimap2 version 2.22 with the parameters “-x ava-ont -r 500,500” (5). Subsequently, we constructed a nondirected graph, with each node representing a read and each edge representing an end-to-end match between two reads; here, a group of densely connected nodes (communities) was considered a set of full-length sequences of each linear plasmid. We identified five linear plasmids (10.4 to 100 kb) and calculated the consensus sequences using the Flye consensus module. Because S1 nuclease removes several nucleotides at the telomere, reads from the non-S1 library were mapped to the assembled chromosome and plasmid sequences to recover the correct telomere ends. A short-read library was prepared from the same gDNA using the NEBNext Ultra II FS DNA library prep kit for Illumina (New England Biolabs) and sequenced using the MiniSeq instrument (Illumina, San Diego, CA) with a 150-bp, paired-end (PE) (300-cycle) reagent. The raw reads were trimmed and quality filtered using fastp version 0.23.2 (6). The assembly was polished using NextPolish version 1.4.0 (7) and Polypolish version 0.5.0 (8) with the short reads (87 Mb). To check the small circular plasmid dropout during the Nanopore ligation-based library preparation (9), the unmapped short reads were collected and *de novo* assembled using MEGAHIT version v1.2.9 (10). No plausible contigs were recovered. Annotation was performed using the DDBJ Fast Annotation and Submission Tool (11). Default parameters were used for all software programs unless otherwise specified.

The 1,195,152-bp linear “*Ca.* Borrelia fainii” genome (GC content, 28.4%) contains 1,096 protein-coding genes. The total sequence coverage was 890× (Nanopore, 818×; MiniSeq, 72×). The 951,861-bp chromosome contains 852 protein-coding genes, 32 tRNAs, and 3 complete rRNAs. The largest linear plasmid, lp100, comprises 100,107 bp and 97 protein-coding genes. The lengths of the linear plasmids lp59, lp42, lp32, and lp10 were 59,167, 42,063, 31,545, and 10,409 bp, respectively. A total of 239 protein-coding genes were present in these five plasmids.

### Data availability.

The sequences are available at DDBJ/GenBank under the accession numbers AP027070, AP027071, AP027072, AP027073, AP027074, and AP027075, and the raw NGS reads are available under the SRA accession numbers DRR424728, DRR424729, DRR424730.
